# The influence of solvent on conformational properties of peptides with Aib residue—a DFT study

**DOI:** 10.1007/s00894-017-3508-4

**Published:** 2017-11-21

**Authors:** Roksana Wałęsa, Małgorzata A. Broda

**Affiliations:** Faculty of Chemistry, University of Opole, 48, Oleska St., 45-052 Opole, Poland

**Keywords:** α-Aminoisobutyric acid residue, *N*-methylation, Conformational analysis, Solvent effect, DFT calculations, X-ray crystallography

## Abstract

The conformational propensities of the Aib residue on the example of two model peptides Ac-Aib-NHMe (**1**) and Ac-Aib-NMe_2_ (**2**), were studied by B3LYP and M06-2X functionals, in the gas phase and in the polar solvents. To verify the reliability of selected functionals, we also performed MP2 calculations for the tested molecules in vacuum. Polarizable continuum models (PCM and SMD) were used to estimate the solvent effect. Ramachandran maps were calculated to find all energy minima. Noncovalent intramolecular interactions due to hydrogen-bonds and dipole attractions between carbonyl groups are responsible for the relative stabilities of the conformers. In order to verify the theoretical results, the available conformations of similar X-ray structures from the Cambridge Crystallographic Data Center (CCDC) were analyzed. The results of the calculations show that both derivatives with the Aib residue in the gas phase prefer structures stabilized by intramolecular N–H⋯O hydrogen bonds, i.e., C_5_ and C_7_ conformations, while polar solvent promotes helical conformation with φ, ψ values equal to +/−60°, +/−40°. In addition, in the case of molecule **2**, the helical conformation is the only one available in the polar environment. This result is fully consistent with the X-ray data.

**Electronic supplementary material:**

The online version of this article (10.1007/s00894-017-3508-4) contains supplementary material, which is available to authorized users.

**Graphical abstract:**
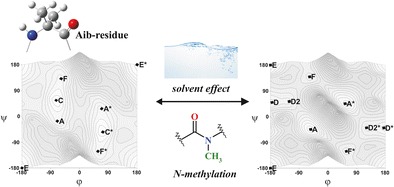
Effect of solvent on the Ramachandran maps of the model peptides with Aib residue

## Introduction

Achiral α-aminoisobutyryl residue (Aib, α,α-dimethylglycine) is a common component in peptides produced by various microorganisms [1–4]. *gem*-Dimethyl substitution on the C^α^-atom severely reduces the conformational freedom of this amino acid residue. Fungal peptides with proven antibiotic activity containing at least one α,α-dimethylglycine residue are called peptaibiotics [5]. Alamethicin and antyameobin [5] were the first examined and characterized peptaibiotics. Moreover, among known peptaibiotics are chlamydocin with cyclic backbone [4], zervamicin and emerimicin [6].

Since the Aib amino acid is not ribosomally encoded, peptides containing this residue are more resistant to proteolytic enzymes than peptides containing protein amino acids only. The Aib residue is used as a modifier of naturally occurring and biologically active peptides [7, 8] due to its unique structural features, introduced by the presence of two methyl groups at C^α^.

Analysis of peptide crystal structures shows that Aib residues favor the formation of 3_10_- or α-helical structures. The type of helix depends strongly on peptide chain length and on the number of the Aib residues in the peptide. So, it is well recognized that tri-, tetra- and pentapeptides containing at least one Aib residue adopt mainly a 3_10_ helix conformation. However, longer (6–20 residues) Aib-containing peptides fold predominantly, but not exclusively, into left- or right-handed α-helices [4, 5, 9]. The vibrational circular dichroism (VCD) and infre-red (IR) methods are especially reliable for discriminating 3_10_- and α-helices [10]. α-Aminoisobutyric acid homooligopeptides in the gas phase and solution were recently studied by Barone and coworkers [11–14] using an improved AMBER force field. In these studies, the solvent effect was shown as the critical factor governing the conformational behavior of a single Aib residue and in homooligopeptides. Molecular dynamics simulations show that the α-helix is the preferred structure in aqueous solution, while in DMSO the 3_10_-helical structure is predominant.

The α,α-dimethylglycine residue also shows a strong tendency, even stronger than that of proline [15], to promote β-turn conformations. For Aib residues, β-turn conformations of type I, I′ and III, III’ are usually observed when this non-standard amino acid residue is placed at both corners of turns. However, occurrence of the Aib residue at the *i + 2* position results in a type II β-turn [15–19]. The peptide with the Aib-Gly turn-initiating sequence shows a very stable β-hairpin conformation over a wide temperature range, as studied by isotope-edited IR spectroscopy and molecular modeling [20, 21].

The conformational properties of the Aib residue have been extensively studied theoretically. The conformational preferences of a model Ac-Aib-NHMe peptide containing the Aib residue were established for the first time in 1972 [22]. According to the latter authors, the α-aminoisobutyryl residue has a strong tendency to adopt helical conformations, and typical torsion angles φ, ψ for the Aib residue are −57° and −47°, respectively. Subsequent theoretical studies have confirmed these reports. Ramachandran maps calculated using the CFF91 force field indicated that this non-standard amino acid adopts an α-helical conformation in model diamide [23]. However, theoretical studies carried out in the gas phase using quantum-mechanical methods (HF, B3LYP and MP2) showed that the Aib residue has a tendency to adopt C_5_ and C_7_ conformations stabilized by intramolecular hydrogen bonds [24, 25].

Similarly, the potential energy surfaces (PES) calculated by the parm96 force field demonstrated that the most preferred structures of Ac-Aib-NHMe are also C_5_ and C_7_ conformers [26]. PCM/B3LYP/6–31 + G(d,p) calculations in solvent showed that the most stable structure in a water environment is the extended conformer C_5_, but the energy of the γ turn structure is only 1 kcal mol^−1^ higher [24].

The conformational properties of Ac-Aib-NMe_2_ diamide have not been studied as extensively as their non-methylated C-terminal amide bond analog. The potential energy surfaces were calculated using molecular mechanics methods [23]. These calculations show that the most stable conformation of this peptide is the α conformation with torsional angles of φ, ψ = 60° and 60°, respectively.

As mentioned earlier, the use of non-standard residues such as Aib could be beneficial in enhancing the biological effects of natural or modified peptides. Another promising way to improve the pharmacological parameters of peptides is their modification by replacing the hydrogen atom of the amide bond by a methyl group—referred to as *N*-methylation. Introduction of a tertiary amide bond into the peptide chain results in a reduction in conformational freedom of the peptide due to steric hindrance [27, 28]. In peptides modified in this way, the tendency to adop a cis-configuration of the amide bond is frequently observed [29–31]. *N*-Methylation is a powerful means of increasing the proteolytic stability [32, 33], membrane permeability (lipophilicity) [34, 35], and bioavailability [36] of natural peptides. There are several examples of modified, *N*-methylated peptides that exhibit much better pharmacokinetic properties [37–39]. Moreover, a few *N*-methylated peptides are currently being evaluated in clinical trials, displaying the promise of *N*-methylation in delivering next generation drugs [40].

The conformational preferences of peptides depend on a delicate balance between intramolecular interactions and the impact of the environment. Although H-bonded interactions are mainly electrostatic in nature, the contribution of dispersion forces in computing accurate interaction energies is not negligible. The hybrid functional B3LYP does not describe dispersion forces correctly [41], and often underestimates the energy of the hydrogen bond [38, 39]. The meta-GGA M06-2X dispersion corrected functional gives better results as regards the energy and geometry of hydrogen bonds [42–45, 69]; however, in some cases, it overestimates the interaction energies and predicts unreasonable structures of N–H⋯O hydrogen bonds in peptides [46]. In this study, we wanted to see how both these methods model the conformational properties of Aib residue derivatives, where we consider dispersive interactions to play a particularly important role.

In this report, we present the results of density functional theory (DFT) calculations on the conformational properties of two model peptides with Aib residues: Ac-Aib-NHMe (**1**) and Ac-Aib-NMe_2_ (**2**). Calculations were performed at the M06-2X/6–31++G(d,p) and B3LYP/6–31++G(d,p) levels of theory in the gas phase, chloroform and water, where the effect of solvent was included using polarized continuum methods (PCM or SMD). We are mainly interested in interactions stabilizing the minima of these compounds, and how the solvent affects their conformational preferences. Another problem analyzed in this paper is the impact of *N*-methylation on the ability of peptides containing Aib residues to adopt typical secondary structure motifs.

## Methods

**Fig. 1:**
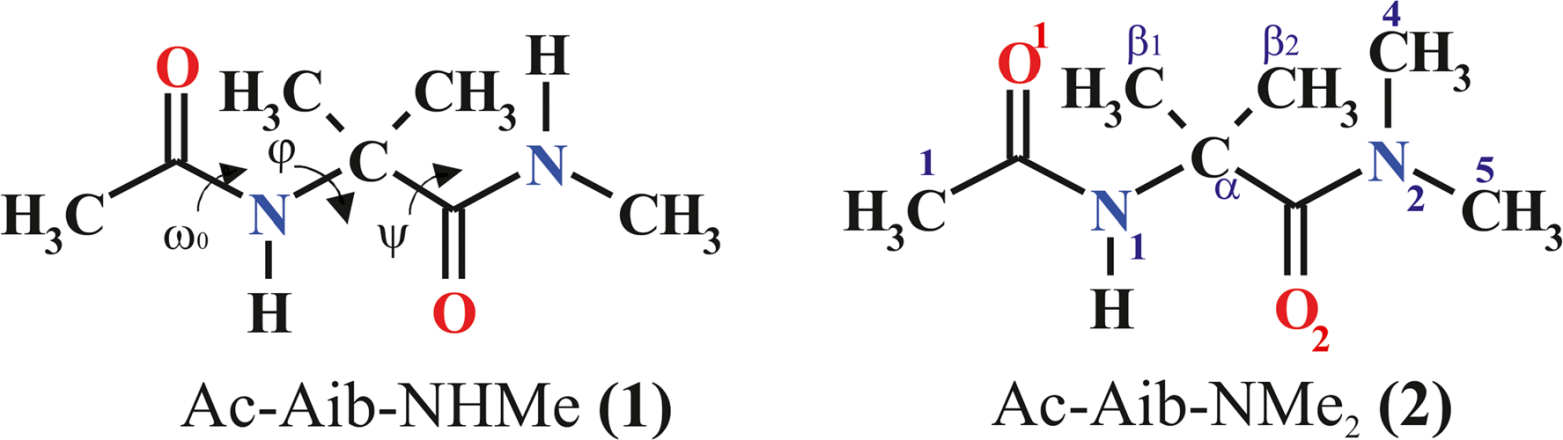
General formula, atom numbering and selected torsion angles of the studied compounds

Theoretical calculations of conformational properties were carried for two model peptides: Ac-Aib-NHMe (**1**) and Ac-Aib-NMe_2_ (**2**) with the *trans N*-terminal amide group (ω_0_ ~ 180°). The chemical structures and torsional parametres of the studied models are defined in Fig. 1. All ab initio and DFT calculations were performed using the Gaussian 09 package [47]. The structural preferences of Ac-Aib-NHMe and Ac-Aib-NMe_2_ were determined by Ramachandran maps E(ϕ,ψ) showing the dependence of potential energy on torsional angles φ and ψ.

Due to the symmetry of the studied molecules, the entire conformational map could be reproduced by calculating only half of the grid points [because E(ϕ,ψ) = E(−ϕ,–ψ)]. The ϕ, ψ PES of each molecule was generated on the basis of 84 structures, partly optimized at the B3LYP/6–31++G(d,p) level. In each grid point, the geometrical parameters were fully relaxed, except for the constrained torsion angles ϕ and ψ. The values of these angles varied from −180° to 0°, and from −180° to 180° for ϕ and ψ, respectively, and the step size were 30°. The energy surface was created using the Surfer 8 program with the radial basis function as a gridding method [48]. Single-point MP2 and M06-2X calculations with 6–31++G(d,p) basis set were performed on partly B3LYP-optimized structures. To estimate the effects of enviroment on the topology of the energy surfaces, single point calculations were conducted for each grid point using two continuum solvent models: PCM [49, 50] and SMD [51].

Ramachandran plots are traditionally used as a convenient way to present the conformational properties of small peptide model, and they are accessible by accurate experimental and computational approaches [23, 24, 26, 30, 52, 53].

All low energy areas of the conformational maps were analyzed, and the minima found were re-optimized using B3LYP, MP2, M06-2X methods with the 6–31++G(d,p) basis set in vacuo. Because we are aware of potential problems of the MP2 approach combined with finite basis sets when applied to conformers of polypeptides [54], for comparison we recalculated the minima using 6–311++G(3df,2pd) basis set. Next, a full geometry optimization in chloroform and water was performed using the PCM and SMD models by B3LYP/6–31++G(d,p) and M06-2X/6–31++G(d,p) methods.

For each conformer, we performed vibrational analysis to check the absence of imaginary freuqencies. The abundances *p* of individual conformers were estimated on the basis of the relative energies [55]. The regions of the Ramachandran map are employed as conformational descriptors for backbone orientations of peptides and are labeled differently by different research groups. In this paper, the energy-minimized conformers of the investigated molecules are described by the general short-hand letter notation introduced by Zimmerman [56].

## Results and discussion

**Fig. 2:**
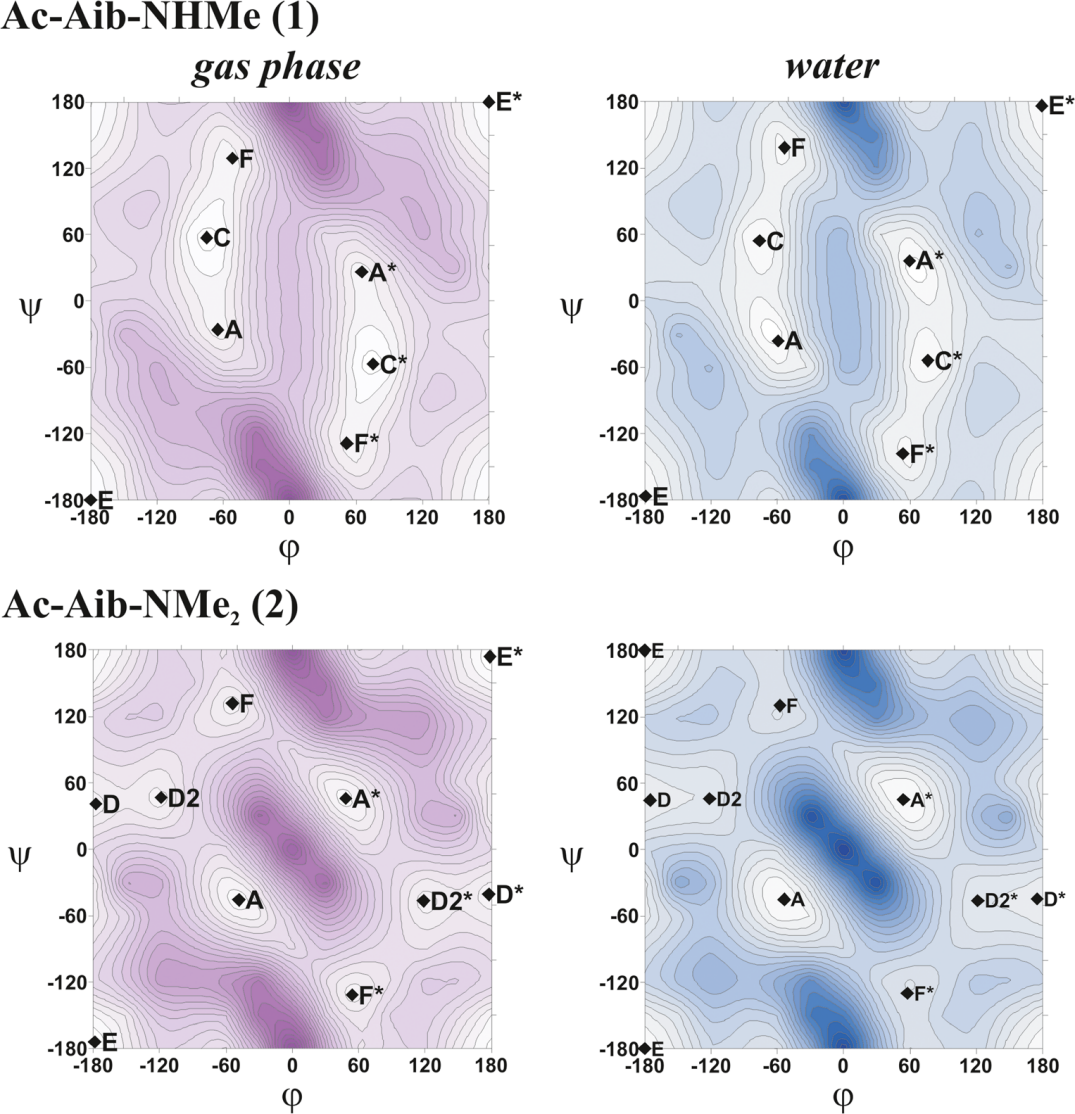
The potential energy surfaces (PES) E = *f*(ϕ, ψ) of Ac-Aib-NHMe (**1**) and Ac-Aib-NMe_2_ (**2**) in vacuo and in water calculated by the M06-2X/6–31++G(d,p) method combined with the polarizable continuum model (PCM) solvent model. Energy contours are drawn every 1 kcal mol^−1^. Local minima are represented by ♦ and described by the general short hand letter notation [44]

Figure 2 presents the ∆E = *f* (φ, ψ) PESs for the studied molecules **1** and **2**, respectively, calculated using the M06-2X method in vacuum and water modeled with the PCM method. The analogous conformational maps calculated in chloroform are presented as Figs. S1 and S2 in the supplementary materials. Also the ∆E(ϕ, ψ) PESs calculated by the MP2 and B3LYP methods in the gas phase and in solvent environments are shown in Figs. S3 and S4 in the supplementary materials. On each map, the local minima are depicted with their Zimmerman notation. Conformations C, E, A, F and D are equivalent to the γ-turn (C_7_), extended (C_5_), α-helical, polyproline-like (P_II_) and β_2_ structures in the literature, respectively. Table 1 lists the backbone torsion angles (ϕ,ψ), relative energies (∆E) and theoretical abundances (*p*) of local minima fully optimized by B3LYP, MP2 and M06-2X methods for studied peptides **1** and **2** in the gas phase. Analogous results of calculations for molecules **1** and **2**, which take into account the effect of the solvent (chloroform or water) are shown in Tables 2 and 3, respectively.

**Table 1:** Selected torsion angles (°), relative energies ∆E (kcal mol^−1^) and theoretical abundances *p* (%) of local minima for (**1**) and (**2**) in vacuo

Conformer	ϕ	ψ	∆E	*p*		ϕ	ψ	∆E	*p*
	Ac-Aib-NHMe (**1**)				Ac-Aib-NMe_2_ (**2**)				
B3LYP/6–31++G(d,p)									
C	−73.0	55.2	0.00	66	E	−180.0	180.0	0.00	99
E	−180.0	−180.0	0.42	32	A	−52.4	−45.2	3.00	1
F	−57.4	126.4	2.59	1	D	−176.8	42.6	3.18	0
A	−69.3	−21.6	2.61	1	D_2_	−118.5	52.7	3.75	0
D	−173.2	34.5	4.50	0	F	−61.1	126.6	3.78	0
M06-2X/6–31++G(d,p)									
C	−75.2	57.0	0.00	62	E	−178.7	−174.1	0.00	80
E	−180.0	−179.9	0.41	31	A	−48.3	−45.8	1.05	14
F	−51.6	128.9	1.67	4	D	−177.5	40.7	2.08	2
A	−65.0	−25.9	1.79	3	F	−54.2	131.9	2.18	2
D	−175.0	35.0	4.02	0	D_2_	−118.5	46.5	2.31	2
MP2/6–31++G(d,p)									
C	−74.3	54.3	0.00	77	E	−179.6	175.2	0.00	45
E	−180.0	−179.9	1.31	9	A	−47.7	−50.6	0.04	42
F	−50.2	131.3	1.38	8	F	−52.1	134.5	1.07	7
A	−63.7	−31.0	1.44	7	D	−178.0	36.7	1.44	4
D	−176.1	33.2	4.26	0	D_2_	−119.0	41.2	2.29	1
MP2/6–311++G(3df,2pd)									
C	−73.7	56.3	0.00	70	E	180.0	−180.0	0.00	73
E	−180.0	−179.9	0.72	21	A	−46.8	−48.5	0.70	22
F	−50.8	132.4	1.61	5	D	−178.6	39.1	2.17	2
A	−63.2	−29.3	1.71	4	F	−53.5	133.5	2.21	2
D	−176.0	32.7	3.65	0	D_2_	−119.0	47.5	2.58	1

**Table 2:** Selected torsion angles (°), relative energies ∆E (kcal mol^−1^) and theoretical abundances *p* (%) of local minima for Ac-Aib-NHNe (**1**) in chloroform or water

Conformer	ϕ	ψ	∆E	*p*		ϕ	ψ	∆E	*p*
	Chloroform				Water				
B3LYP/PCM									
C	−73.6	54.6	0.00	49	A	−62.9	−33.8	0.00	48
E	−180.0	−180.0	0.15	38	C	−73.8	53.5	0.48	22
A	−66.3	−28.8	0.97	10	E	−179.8	−177.7	0.48	22
F	−56.9	135.9	1.57	3	F	−56.7	139.2	1.04	8
D	−171.8	36.7	3.52	0	D	−170.7	36.2	3.26	0
B3LYP/SMD									
E	−179.8	−177.1	0.00	43	F	−54.9	140.0	0.00	54
C	−73.3	53.5	0.08	38	A	−59.8	−35.7	0.29	33
A	−65.3	−28.6	0.76	12	E	−179.4	−179.9	1.02	10
F	−57.0	137.0	1.10	7	C	−74.1	48.2	1.60	4
D	−171.9	38.4	3.26	0	D	−170.4	39.3	3.79	0
M062X/PCM									
C	−75.9	57.3	0.00	36	A	−59.5	−36.1	0.00	76
A	−62.1	−32.4	0.12	30	F	−53.3	138.5	1.19	10
E	−179.2	−176.4	0.25	24	C	−76.3	54.1	1.38	8
F	−52.2	135.8	0.79	10	E	179.2	−176.5	1.54	6
D	−173.3	36.8	3.17	0	D	−172.2	36.9	3.88	0
M062X/SMD									
A	−62.0	−32.5	0.00	33	F	−51.8	140.3	0.00	58
C	−75.7	54.5	0.14	26	A	−56.8	−37.1	0.23	39
E	−178.5	−177.3	0.17	25	E	−179.1	−179.8	2.09	2
F	−52.4	137.2	0.43	16	C	−76.5	48.7	2.47	1
D	−173.3	38.5	3.01	0	D	−170.7	38.2	4.45	0

**Table 3:** Selected torsion angles (°), relative energies ∆E (kcal mol^−1^) and theoretical abundances *p* (%) of local minima for Ac-Aib-NMe_2_ (**2**) in chloroform or water

Conformer	ϕ	ψ	∆E	*p*		ϕ	ψ	∆E	*p*
	Cchloroform				Water				
B3LYP/PCM									
E	−180.0	180.0	0.00	82	A	−53.9	−45.0	0.00	76
A	−54.4	−43.6	0.98	16	E	−180.0	−180.0	0.41	22
D	−175.7	44.2	2.30	2	D	−175.1	44.6	2.55	1
D_2_	−120.2	48.2	3.26	0	D_2_	−121.2	46.0	4.12	0
F	−59.1	128.6	3.61	0	F	−57.5	130.1	4.19	0
B3LYP/SMD									
E	179.3	175.2	0.00	68	A	−53.0	−47.6	0.00	95
A	−55.2	−42.6	0.53	28	E	−179.5	175.8	1.92	4
D	−173.8	45.7	1.93	3	D	−168.6	47.1	3.37	1
D_2_	−121.2	47.2	2.89	1	F	−54.9	138.8	4.26	0
F	−58.1	129.4	3.36	0	D_2_	−121.6	46.2	4.31	0
M06-2X/PCM									
A	−49.5	−44.1	0.00	78	A	−49.4	−44.6	0.00	98
E	−178.4	−179.2	0.87	18	E	−180.0	179.4	2.43	1
F	−54.2	131.1	2.92	1	D	−175.2	43.9	2.43	0
D_2_	−118.7	44.2	2.64	1	D_2_	−118.5	43.0	3.94	0
D	−175.9	43.3	2.24	2	F	−54.4	131.5	3.21	0
M06-2X/SMD									
A	−51.0	−39.8	0.00	85	A	−45.3	−50.1	0.00	100
E	−179.7	−174.6	1.21	11	E	−176.2	−171.4	4.42	0
D	−175.2	44.3	2.21	2	F	−53.8	136.3	4.16	0
D_2_	−118.6	43.8	2.56	1	D_2_	−117.2	41.8	5.08	0
F	−53.8	131.5	2.91	1	D	−168.0	46.9	4.38	0

**Fig. 3:**
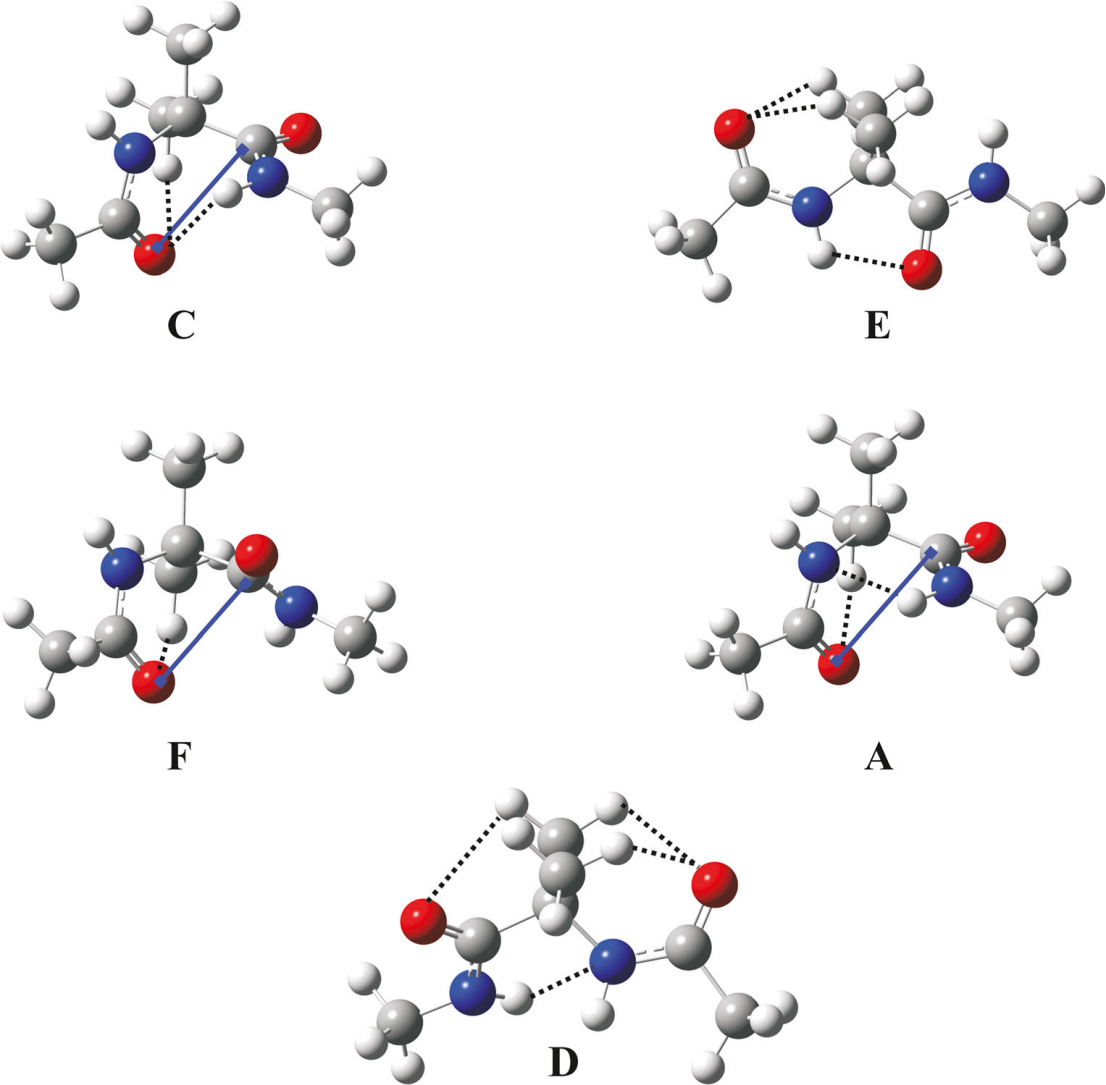
Local minima for Ac-Aib-NHMe (**1**) optimized at the M06-2X/6–31++G(d,p) level in the gas phase. *Dotted lines* Hydrogen bonds, *solid lines* dipole interactions

**Fig. 4:**
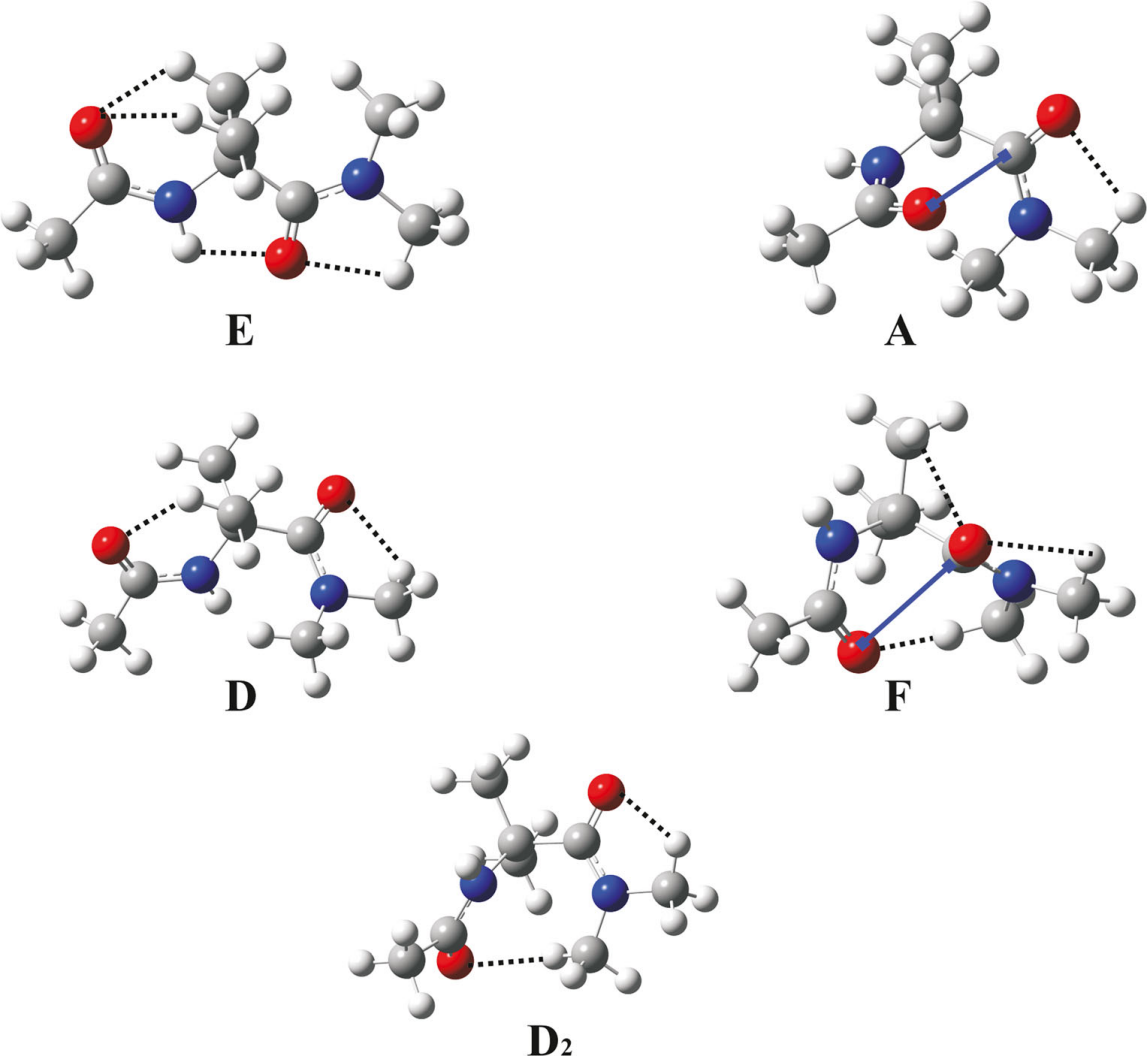
Local minima for Ac-Aib-NMe_2_ (**2**) optimized at the M06-2X/6–31++G(d,p) level in the gas phase. *Dotted lines* Hydrogen bonds, *solid lines* dipole interactions

The presence of achiral α-carbon of the studied molecules results in symmetry of their maps with respect to the point (φ, ψ = 0°, 0°). Therefore, in a discussion of the obtained results, only the minima found in the left halves of the maps have been taken into consideration. A detailed conformational analysis of the conformers of studied molecules was performed on an assumption that hydrogen bonds (N–H⋯O, N–H⋯N, C–H⋯O) and dipole–dipole attractions between carbonyl groups are the main stabilizing internal forces. Tables S1 and S2 in supplementary material collect structural parameters of the X–H⋯A interactions and dipole attractions based on Steiner’s [57] and Allen’s [58] criteria, respectively. Figures 3 and 4 show the local minima with stabilizing interactions for the two studied compounds in the gas phase.

### Ac-Aib-NHMe (**1**)

As shown in Fig. 2, in the gas phase and in water, the M06-2X conformational maps of Ac-Aib-NHMe reveal four low-energy conformers—C, E, F, A—and their mirror images. Moreover, a high energy minimum D (in the gas phase ΔE ≈ 4 kcal mol^−1^ as predicted by M06-2X method) also occurs on Ramachandran maps of molecule **1**, which, due to its energy, probably has no practical significance. The conformational maps obtained with B3LYP and MP2 methods (Fig. S3in supplementary material) look very similar in terms of the shape, number and position of the minima. This probably means that the conformational properties of this compound are determined primarily by steric interactions and that only two areas of the map are accessible: an area of extended conformations with minimum E, and a second area closer to the center of the map, with remaining three low energy minima.

Inspection of the results for molecule **1** listed in Table 1 shows that results obtained by all computational methods used are quite similar. All methods predict that, in a vacuum, both conformers stabilized by N–H⋯O hydrogen bonds (C and E) have low energies (ΔE < 1.4 kcal mol^−1^) and their theoretically estimated abundance is in the range 85–98%. According to the results of all methods, the global minimum of **1** is the conformer C stabilized by relatively short C_7_ N^2^–H⋯O^1^ hydrogen bond ( H⋯O distance =1.93 Å) (see Table S1 in supplementary material) and one weak C^β^–H⋯O^1^ interaction (Fig. 3). Moreover, dipole attraction between the two carbonyl groups also stabilizes this conformation.

The second low energy conformer, E, shows M06-2X relative energy of about 0.4 kcal mol^−1^ higher. This conformer is a fully extended structure with torsion angles φ, ψ **=** −180°, −179°, and is stabilized mainly by the C_5_ hydrogen bond N^1^–H⋯O^2^. Additionally, two hydrogen bonds C^β^–H⋯O^1^ seem to play role in the stabilization of this conformation. The relative energies of conformer E calculated by M06-2X and B3LYP methods are essentially identical. However, the relative energy of this structure obtained with MP2 method is much higher (∆E = 1.31 kcal mol^−1^). Overstated energy for extended structures obtained by teh MP2 method with this double zeta basis set was also reported for Ac-Gly-Phe-NH_2_, Ac-Gly-ΔPhe-NHMe and Ac-Gly-ΔPhe-NMe_2_ dipeptides [59, 60]. With the use of a larger triple zeta basis set [6–311++G(3df,2pd)] within the MP2 method, this effect was no longer observed.

Moreover, it is worth noting that both DFT methods estimate the stability of helical conformer A differently. This conformer (φ, ψ = −65°, −26°) is non N–H⋯O hydrogen-bonded. The main stabilizing force is a weak N^2^–H⋯N^1^ interaction, which is present only in this conformer and C^β^–H⋯O^1^ contact (see Table S1 in supplementary material). It also has a short antiparallel dipole–dipole attraction (see Table S2). It is interesting that the B3LYP results indicate the high energy of conformer A in the gas phase (ΔE ≈ 2.6 kcal mol^−1^). The results obtained using the M06-2X method (ΔE ≈ 1.8 kcal mol^−1^) seem more reliable because MP2/6–311++G(3df,2pd) predicts similar relative energies of the obtained conformers. Furthermore, this is also consistent with the experimentally demonstrated ability of the Aib residue to induce an α-helix conformation of the peptide [4, 5, 9].

A systematic exploration of the potential energy surface and full geometry optimizations for all local minima obtained in the gas phase were also carried out in chloroform and water using M06-2X and B3LYP methods, combined with PCM and SMD models (Table 2). Calculations predict that the solvent effect on the conformational properties of Ac-Aib-NHMe is rather limited. As already mentioned, the topology of maps calculated in a vacuum and taking into account the effect of the solvent are very similar. All local minima found in the gas phase are also present in chloroform and water, and conformer D always has a high energy (ΔE > 3 kcal mol^−1^). However, the transition from the gas phase to chloroform and water causes some shifts in the backbone torsion angles φ, ψ for the local minima. In particular, the largest changes were observed for conformations A and F, the backbone dihedral angles of which correspond to the α-helix and polyproline II helix (PII) conformations, respectively. For example, on going from the gas phase to water, the M06-2X method combined with PCM model provides shifts of −10.2° and +9.6° in ψ of the conformation A and F, respectively. The corresponding values are −14.1° and +13.6° at the SMD/B3LYP/6–31++G(d,p) level.

Chloroform and water significantly reduce the energy differences between the minima. For example, in chloroform the gap energy between the first and the fourth conformer equals 0.43 kcal mol^−1^ in the case of geometries calculated at M06-2X level with SMD model of solvent, and 0.79 kcal mol^−1^ with PCM. Furthermore, solvent environment rearranges the order of low-energy minima. Besides, for each method, different global minima were obtained, and their energies differ less than the error of the DFT methods used. However, a general regularity may be noted: both DFT methods and both solvent models predict a significant stabilization of helical conformer A. Calculations with PCM model predict that conformer A, with torsion angles φ, ψ = −60°, −36°, becomes the global minimum in water. Conformer A is also the most preferred structure of the studied molecule in chloroform at the SMD M06-2X/6–31++G(d,p) level of theory. Another conformer strongly stabilized by the solvent is structure F. Thus, the results obtained by the SMD method predict that conformer F is the lowest-energy structure in water.

It is worth noting that these two conformations discussed above (A and F), are the most strongly influenced by the polar environment, and are stabilized mainly by the short and strong dipole C = O attractions between the carbonyls of the amide groups (see Table S2). The theoretically estimated abundances of these two conformers in water using PCM/M06-2X and SMD/M06-2X models are 86% and 97%, respectively.

The above-described effect of solvent on conformational properties of Ac-Aib-NHMe is very similar to that obtained for the most studied Ac-Ala-NHMe model peptide. Various ab initio and DFT calculations indicated that solvent effects stabilize the conformer corresponding to the α-helix secondary structure, and flatten the PES [61–63]. The results of explicit water calculations also show that hydration of the peptide backbone critically depends on the backbone conformation, and allowed us to determine that the N–H⋯O H-bond formed by a dipeptide in its extended conformation is weakened by the close proximity of the O atom of the neighboring peptide group to the NH proton donor [46, 63–68].

### Ac-Aib-NMe_2_ (**2**)

Table 1 shows the backbone torsion angles and relative energies of the local minima obtained for Ac-Aib-NMe_2_. The conformational maps of this molecule in the gas phase, chloroform and water reveal five local minima—A, E, F, D_2_, D—and their mirror images (Fig. 2) regardless of calculation method (the conformational maps obtained with B3LYP and MP2 methods are presented at Fig. S4 in supplementary material). The presence of an additional methyl group at the C-terminal amide bond results in increasing the energy of potential surface center, which corresponds to conformation with φ, ψ = 0°,0° torsion angles. This is caused by a steric repulsion between the C-terminal methyl group (−C^5^H_3_) and the oxygen atom of the C = O^1^ group, and instead of the C_7_ conformation observed for Ac-Aib-NHMe (**1**), there are two high-energy local D minima stabilized by two weak C–H⋯O contacts.

In the gas phase, the most preferred conformation found by both DFT (M06-2X, B3LYP) and MP2 methods, is the fully extended structure situated in region E (φ, ψ torsion angles are almost 180°). In this conformation, the short C_5_ N^1^–H⋯O^2^ hydrogen bond is the main stabilizing factor, and the H⋯O^2^ distance, according to the M06-2X functional, is only 1.76 Å. Moreover, there are three C–H⋯O contacts: C^5^–H⋯O^2^, C^β1^–H⋯O^1^, C^β2^–H⋯O^1^, which additionally stabilize this global minimum (Fig. 4).

All calculations predict that the second in energy order is conformer A, stabilized mainly by dipole–dipole interaction between C = O groups and by one weak C^4^–H⋯O^2^ hydrogen bond. Its relative energy depends strongly on the method of calculation. As in the case of molecule (**1**), B3LYP density functional distinctly overestimates energy of this helical conformation. A more reliable result is obtained with M06-2X (ΔE = 1.05 kcal mol^−1^) because this value is closer to that obtained using the MP2/6–311++G(3df,2pd) method.

The conformational preferences of Ac-Aib-NMe_2_ (**2**) were examined also in chloroform and water (Table 3, Fig. 1). For neither solvent, did we observe a significant effect on the conformation of the compound. The shape of the maps and the number and location of the minima are the same as in the gas phase. However, in all cases, we observe a clear stabilization of the helical conformation A. Regardless of the calculation method and model of solvent, the two lowest-energy structures are conformers A and E, and their summary theoretically estimated abundance is 96–98%. Increased solvent polarity stabilizes the helical conformation to a greater extent. All our results indicate that, in water, this conformer is a global minimum, which remains in accordance with experimental data.

### Solvent effects on conformation

**Fig. 5:**
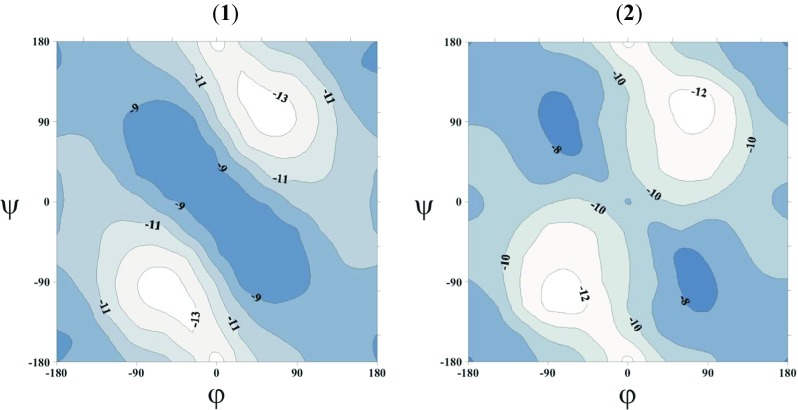
Solvation energies of the studied molecules Ac-Aib-NHMe (**1**), Ac-Aib-NMe_2_ (**2**) in water as a function of backbone conformation (φ and ψ values) obtained using the PCM/M06-2X/6–31++G(d,p) method

Figure 5 shows the energy of the interaction with water (estimated within the implicit solvent model PCM) as a function of the torsion angles φ, ψ for both studied molecules. The brightest areas on the maps indicate conformations of molecules **1** and **2** with the most significant solvent influence. As can be seen, for both compounds, the highest solvent stabilization energies are observed for helical conformations. The energy maxima occur at (ϕ,ψ = (−60°, −100°) and (−70°, −110°) for **1** and **2**, respectively. These results are similar to those obtained for Ac-Ala-NHMe with a trans N-terminal amide bond [61, 68]. The presence of an additional methyl group at the C-terminal part in diamide **2** causes slightly weaker interaction with the solvent, by about 1 kcal mol^−1^. Additionally, the results of calculations show that there is a large difference in the energy values obtained by the PCM and SMD models. Within the SMD model, energy values are 5 kcal mol^−1^ and 6 kcal mol^−1^ higher for chloroform and water, respectively. Despite these differences, we can conclude that the solvent stabilizes helical conformations of the studied Aib derivatives, and that this is closely related to the dipole moment of those compounds [30].

### X-ray structures of Aib derivatives

**Fig. 6:**
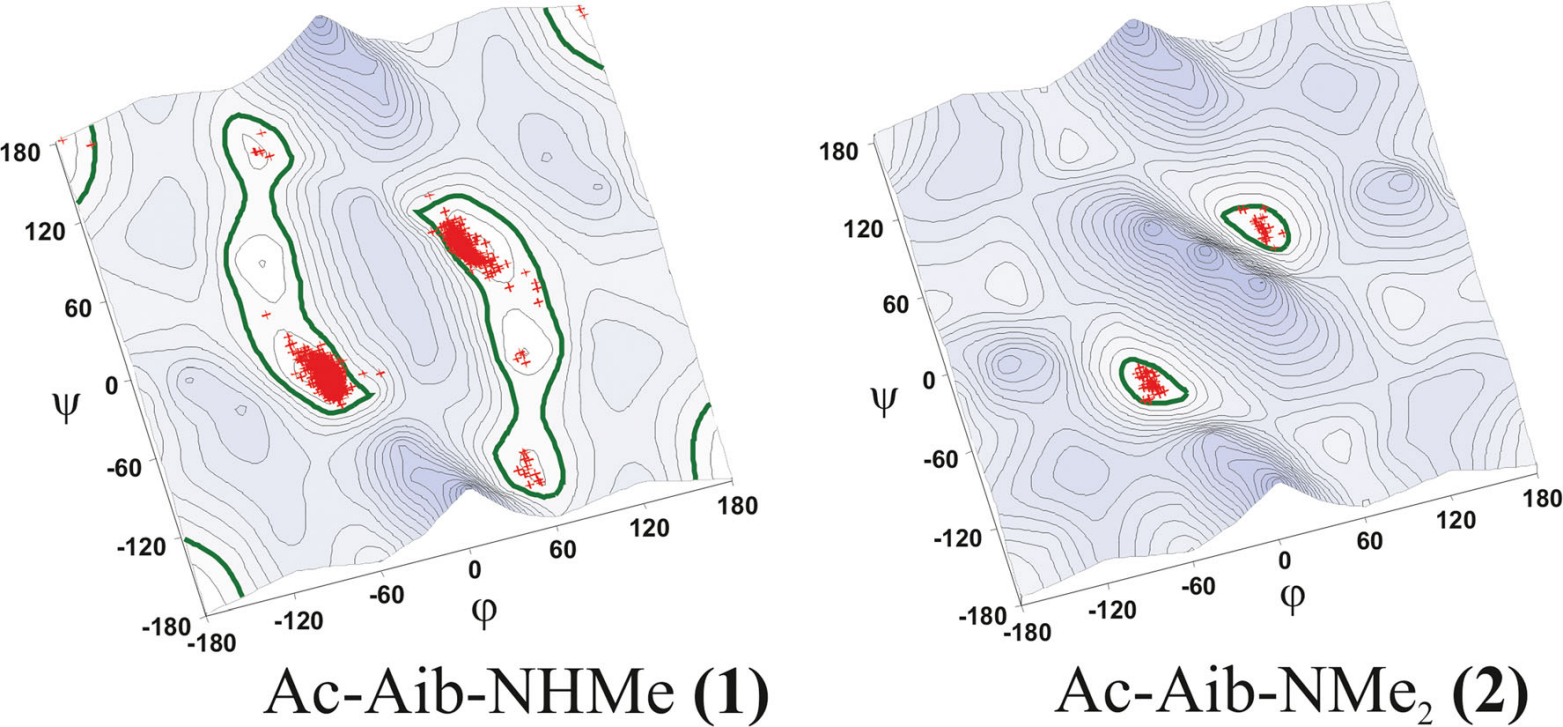
The ϕ, ψ potential energy surfaces (PESs) of the studied molecules in water with the solid state conformations of analogous structures (*crosses*) from Cambridge Structural Database. *Bold line* Energy contour 4 kcal mol^−1^, showing accessible conformational space: 15% for **1**, 4% for **2**

To verify the obtained theoretical results, the conformations of the peptides containing the Aib residue, gathered in the Cambridge Crystallographic Data Center (CCDC), were analyzed. The database search yielded 1116 peptide structures with the Aib residue in the vicinity of the secondary amide bond, and 68 structures with a tertiary amide bond. On the calculated map of molecules **1** and **2** in water the φ, ψ torsion angles values corresponding to the conformers found in the crystal state of Aib derivatives are marked with red crosses (Fig. 6). It is apparent from the maps presented that practically all the X-ray structures from CCDC are in regions of the calculated minima. For molecule **1**, the vast majority of them are structures, where the Aib residues adopt the right or left-handed helix conformation, 69% and 29%, respectively, while 1.7% of all crystal structures correspond to structures F. For Aib residues in the vicinity of the tertiary amide bond, all crystal structures found are within the helical conformation (62% right-handed helix, 38% left-handed helix).

On each map (Fig. 6) the green bold line defines the area of conformations with relative energies < 4 kcal mol^−1^. Determined in this way, available areas of Ramachandran diagram for diamides **1** and **2** are 15% and 4%, respectively. This means that the tertiary amide group at the C-terminal side of Aib residue significantly reduces the conformational freedom of the compound.

## Conclusions

The results of the theoretical calculations presented in this paper highlight the effect of chloroform and water on the conformational properties of model peptides with Aib residue. The conformational preferences of two Aib derivatives Ac-Aib-NHMe (**1**) and Ac-Aib-NMe_2_ (**2**) have been explored by the M06-2X/6–31++G(d,p) and B3LYP/6–31++G(d,p) methods in the gas phase and in a solvent environment, and by ab initio calculations at the MP2/6–311++G(3df,2pd) level in the gas phase. The results obtained show that both studied model peptides in the gas phase adopt structures stabilized by N–H⋯O hydrogen bonds, i.e., C_5_ or C_7_ conformations. However, in the polar environment, helical conformations φ, ψ = (+/− 60°, +/− 40°) are the most stable, especially in the case of Aib residues in the vicinity of the tertiary amide. As a result, in the case of molecule **2**, the helical conformation is the only one available in the polar solvent. These conclusions fully agree with the crystallographic data. The CCDC data shows that 98% of structures -Aib-NH- and 100% of -Aib-NMe- are in helical conformation.

## Electronic supplementary material

ESM 1 (PDF 3.71 mb).

## References

[1] De Zotti M, Biondi B, Park Y, Hahm KS, Crisma M, Toniolo C, Formaggio F (2012). Antimicrobial lipopeptaibol trichogin GA IV: Role of the three Aib residues on conformation and bioactivity. Amino Acids.

[2] Weigelt S, Huber T, Hofmann F, Jost M, Ritzefeld M, Luy B, Freudenberger C, Majer S, Vass E, Greie JC, Panella L, Kaptein B, Broxterman QB, Kessler H, Altendorf K, Hóllsi M, Sewald N (2012). Synthesis and conformational analysis of Efrapeptins. Chem Eur J.

[3] Karle IL (1999). Aspects of peptide folding and aggregation. Acc Chem Res.

[4] Karle IL, Balaram P (1990). Structural characteristics of α-helical peptide molecules containing Aib residues. Biochemistry.

[5] Aravinda S, Shamala N, Balaram P (2008). Aib residues in Peptaibiotics and synthetic sequences: Analysis of nonhelical conformations. Chem Biodivers.

[6] Rinehart KL, Gaudioso LA, Moore ML, Pandey RC, Cook JC, Barber M, Sedgwick RD, Bordoli RS, Tyler AN, Green BN (1981). Structures of eleven zervamicin and two emerimicin peptide antibiotics studied by fast atom bombardment mass spectrometry. J Am Chem Soc.

[7] Frydman-Marom A, Convertino M, Pellarin R, Lampel A, Shaltiel-Karyo R, Segal D, Caflisch A, Shalev DE, Gazit E (2011). Structural basis for inhibiting β-amyloid oligomerization by a non-coded β-breaker-substituted endomorphin analogue. ACS Chem Biol.

[8] Conlon JM, Al-Kharrge R, Ahmed E, Raza H, Galadari S, Condamine E (2007). Effect of aminoisobutyric acid (Aib) substitutions on the antimicrobial and cytolytic activities of the frog skin peptide temporin-1Dra. Peptides.

[9] Gessmann R, Brückner H, Petratos K (2016). The crystal structure of Z-(Aib)_10_-OH at 0.65 Å resolution: three complete turns of 3_10_-helix. J Pept Sci.

[10] Silva RAGD, Yasui SC, Kubelka J, Formaggio F, Crisma M, Toniolo C, Keiderling TA (2007). Discriminating 3_10_- from α-helices: vibrational and electronic CD and IR absorption study of related Aib-containing oligopeptides. J Am Chem Soc.

[11] Improta R, Rega N, Aleman C, Barone V (2001). Conformational behavior of macromolecules in solution. Homopolypeptides of α-aminoisobutyric acid as test cases. Macromolecules.

[12] Improta R, Barone V, Kudin KN, Scuseria GE (2001). Structure and conformational behavior of biopolymers by density functional calculations employing periodic boundary conditions. I. The case of polyglycine, polyalanine, and poly-α-aminoisobutyric acid in vacuo. J Am Chem Soc.

[13] Grubišić S, Chandramouli B, Barone V, Brancato G (2016). Chain length, temperature and solvent effects on the structural properties of α-aminoisobutyric acid homooligopeptides. Phys Chem Chem Phys.

[14] Grubišić S, Brancato G, Barone V (2013). An improved AMBER force field for α,α-dialkylated peptides: intrinsic and solvent-induced conformational preferences of model systems. Phys Chem Chem Phys.

[15] Byun BJ, Song IK, Chung YJ, Ryu KH, Kang YK (2010). Conformational preferences of X-pro sequences: Ala-pro and Aib-pro motifs. J Phys Chem B.

[16] Ro S, Lee HJ, Ahn IA, Shin DK, Lee KB, Yoond CJ, Choie YS (2001). Torsion angle based design of peptidomimetics: a dipeptidic template adopting β-I turn (ac-Aib-AzGly–NH_2_). Bioorg Med Chem.

[17] Raghothama S, Chaddha M, Balaram P (1996). Determination of Aib residue conformation in peptides using diagnostic sidechain-backbone nuclear Overhauser effects. Proc Natl Acad Sci India.

[18] Masterson LR, Etienne MA, Porcelli F, Barany G, Hammer RP, Veglia G (2007). Nonstereogenic α-aminoisobutyryl-glycyl dipeptidyl unit nucleates type I’ β-turn in linear peptides in aqueous solution. Biopolymers.

[19] Mahalakshmi R, Balaram P, Guerois R, dela Paz López M (2006). Non-protein amino acids in the design of secondary structure scaffolds. Methods in molecular biology. Protein design: methods and applications, vol 340.

[20] Huang R, Setnička V, Etienne MA, Kim J, Kubelka J, Hammer RP, Keiderling TA (2007). Cross-strand coupling of a β-hairpin peptide stabilized with an Aib-Gly turn studied using isotope-edited IR spectroscopy. J Am Chem Soc.

[21] Kim J, Huang R, Kubelka J, Bouř P, Keiderling TA (2006). Simulation of infrared spectra for β-hairpin peptides stabilized by an Aib-Gly turn sequence: correlation between conformational fluctuation and vibrational coupling. J Phys Chem B.

[22] Marshall GR, Bosshard HE (1971). Angiotensin II. Studies on the biologically active conformation. Circ Res.

[23] Tran TT, Treutlein H, Burgess AW (2006). Designing amino acid residues with single- conformations. Protein Eng Des Sel.

[24] Casanovas J, Zanuy D, Nussinov R, Alemán C (2007). Intrinsic conformational characteristics of α,α-diphenylglycine. J Organomet Chem.

[25] Alemán C (1997). Conformational properties of r-amino acids disubstituted at the α-carbon. J Phys Chem B.

[26] Bisetty K, Catalan JG, Kruger HG, Perez JJ (2005). Conformational analysis of small peptides of the type ac–X–NHMe, where X=Gly, ala, Aib and cage. J Mol Struct.

[27] Goodfellow VS, Marathe MV, Kuhlman KG, Fitzpatrick TD, Cuadrado D, Hanson W, Zuzack JS, Ross SE, Wieczorek M, Burkard M, Whalley ET (1996). Bradykinin receptor antagonists containing *N*-substituted amino acids: in vitro and in vivo B2 and B1 receptor antagonist activity. J Med Chem.

[28] Heller M, Sukopp M, Tsomaia N, John M, Mierke DF, Reif B, Kessler H (2006). The conformation of cyclo(−d-pro-Ala4-) as a model for cyclic pentapeptides of the DL4 type. J Am Chem Soc.

[29] Bágyi I, Balogh B, Czajlik A, Éliás O, Gáspári Z, Gergely V, Hudáky I, Hudáky P, Kalászi A, Károlyházy L, Keserû K, Kiss R, Krajsovszky G, Láng B, Nagy T, Rácz Á, Szentesi A, Tábi T, Tapolcsányi P, Vaik J, Koo JCP, Chass GA, Farkas Ö, Perczel A, Mátyus P (2003). Generation and analysis of the conformational potential energy surfaces of N-acetyl-N-methyl-l-alanine-N′-methylamide. An exploratory ab initio study. J Mol Struct.

[30] Wałęsa R, Broda MA (2014). Solvent effects on the conformational preferences of model peptoids. MP2 study. J Pept Sci.

[31] Trzepałka E, Kowalczyk W, Lammek B (2004). Cis/trans conformational equilibrium across the *N*-methylphenylalanine 2-N-methylphenylalanine3 peptide bond of arginine vasopressin analogs. J Pept Res.

[32] Chatterjee J, Gilon C, Hoffman A, Kessler H (2008). *N*-Methylation of peptides: a new perspective in medicinal chemistry. Acc Chem Res.

[33] Biron E, Chatterjee J, Ovadia O, Langenegger D, Brueggen J, Hoyer D, Schmid HA, Jelinek R, Gilon C, Hoffman A, Kessler H (2008). Improving oral bioavailability of peptides by multiple N-methylation: Somatostatin analogues. Angew Chem Int Ed Eng.

[34] Gordon DJ, Tappe R, Meredith SC (2002). Design and characterization of a membrane permeable N-methyl amino acid-containing peptide that inhibits Aβ1-40 fibrillogenesis. J Pept Res.

[35] Ovadia O, Greenberg S, Laufer B, Gilon C, Hoffman A, Kessler H (2010). Improvement of drug-like properties of peptides: the somatostatin paradigm. Expert Opin Drug Discovery.

[36] Cody WL, He JX, Reily MD, Haleen SJ, Walker DM, Reyner EL, Stewart BH, Doherty AM (1997). Design of a potent combined pseudopeptide endothelin-a/endothelin-b receptor antagonist, ac-dBhg^16^-Leu-asp-IIe-[NMe]IIe-Trp^21^ (PD 156252): examination of its pharmacokinetic and spectral properties. J Med Chem.

[37] Harris KS, Casey JL, Coley AM, Karas JA, Sabo JK, Tan YY, Dolezal O, Norton RS, Hughes AB, Scanlon D, Foley M (2009). Rapid optimization of a peptide inhibitor of malaria parasite invasion by comprehensive N-methyl scanning. J Biol Chem.

[38] Rajeswaran WG, Hocart SJ, Murphy WA, Taylor JE, Coy DH (2001). N-methyl scan of somatostatin octapeptide agonists produces interesting effects on receptor subtype specificity. J Med Chem.

[39] Haviv F, Fitzpatrick TD, Swenson RE, Nichols CJ, Mort NA, Bush EN, Diaz G, Bammert G, Nguyen A, Rhutasel NS, Nellans HN, Hoffman DJ, Johnson ES, Greer J (1993). Effect of *N*-methyl substitution of the peptide bonds in luteinizing hormone-releasing hormone agonists. J Med Chem.

[40] Chatterjee J, Rechenmacher F, Kessler H (2013). *N*-Methylation of peptides and proteins: an important element for modulating biological functions. Angew Chem Int Ed.

[41] Van Mourik T (2008). Assessment of density functionals for intramolecular dispersion-rich interactions. J Chem Theory Comput.

[42] Burns LA, Vázquez-Mayagoitia A, Sumpter BG, Sherrill CD (2011). Density-functional approaches to noncovalent interactions: a comparison of dispersion corrections (DFT-D), exchange-hole dipole moment (XDM) theory, and specialized functionals. J Chem Phys.

[43] Zhao Y, Truhlar DG (2011). Applications and validations of the Minnesota density functionals. Chem Phys Lett.

[44] Thanthiriwatte KS, Hohenstein EG, Burns LA, Sherrill CD (2011). Assessment of the performance of DFT and DFT-D methods for describing distance dependence of hydrogen-bonded interactions. J Chem Theory Comput.

[45] Dilabio GA, Johnson ER, Otero-De-La-Roza A (2013). Performance of conventional and dispersion-corrected density-functional theory methods for hydrogen bonding interaction energies. Phys Chem Chem Phys.

[46] Buczek A, Broda MA (2014). DFT study of N–H⋯O hydrogen bond between model dehydropeptides and water molecule. Mol Phys.

[47] Gaussian 09, Revision E.01, Frisch MJ, Trucks GW, Schlegel HB, Scuseria GE, Robb MA, Cheeseman JR, Scalmani G, Barone V, Mennucci B, Petersson GA, Nakatsuji H, Caricato M, Li X, Hratchian HP, Izmaylov AF, Bloino J, Zheng G, Sonnenberg JL, Hada M, Ehara M, Toyota K, Fukuda R, Hasegawa J, Ishida M, Nakajima T, Honda Y, Kitao O, Nakai H, Vreven T,. Montgomery Jr JA, Peralta JE, Ogliaro F, Bearpark M, Heyd JJ, Brothers E, Kudin KN, Staroverov VN, Keith T, Kobayashi R, Normand J, Raghavachari K, Rendell A, Burant JC, Iyengar SS, Tomasi J, Cossi M, Rega N, Millam JM, Klene M, Knox JE, Cross JB, Bakken V, Adamo C, Jaramillo J, Gomperts R, Stratmann RE, Yazyev O, Austin AJ, Cammi R, Pomelli C, Ochterski JW, Martin RL, Morokuma K, Zakrzewski VG, Voth GA, Salvador P, Dannenberg JJ, Dapprich S, Daniels AD, Farkas O, Foresman JB, Ortiz JV, Cioslowski J, Fox DJ (2013) Gaussian, Inc. Wallingford CT

[48] Surfer 8, Golden Software Inc. (2002) http://www.goldensoftware.com/products/surfer

[49] Miertus S, Tomasi J (1982). Approximate evaluations of the electrostatic free energy and internal energy changes in solution processes. J Chem Phys.

[50] Tomasi J, Mennucci B, Cammi R (2005). Quantum mechanical continuum solvation models. Chem Rev.

[51] Marenich AV, Cramer CJ, Truhlar DG (2009). Universal solvation model based on the generalized born approximation with asymmetric descreening. J Phys Chem B.

[52] Vymětal J, Vondrášek J (2010). Metadynamics as a tool for mapping the conformational and free-energy space of peptides—the alanine dipeptide case study. J Phys Chem B.

[53] Parchaňský V, Kapitán J, Kaminský J, Šebestík J, Bouř P (2013). Ramachandran plot for alanine dipeptide as determined from Raman optical activity. J Phys Chem Lett.

[54] Goerigk L, Karton A, Martin JML, Radom L (2013). Accurate quantum chemical energies for tetrapeptide conformations: why MP2 data with an insufficient basis set should be handled with caution. Phys Chem Chem Phys.

[55] Hudáky I, Perczel A (2008). Prolylproline unit inmodel peptides and in fragments from databases. Proteins Struct Funct Genet.

[56] Zimmerman SS, Pottle MS, Némethy G, Scheraga HA (1977). Conformational analysis of the 20 naturally occurring amino acid residues using ECEPP. Macromolecules.

[57] Steiner T (2002). The hydrogen bond in the solid state. Angew Chem Int Ed.

[58] Allen FH, Baalham CA, Lommerse JPM, Raithby PR (1998). Carbonyl-carbonyl interactions can be competitive with hydrogen bonds. Acta Crystallogr B.

[59] Sǎrič A, Hrenar T, Mališ M, Došlič N (2010). Quantum mechanical study of secondary structure formation in protected dipeptides. Phys Chem Chem Phys.

[60] Buczek A, Wałęsa R, Broda MA (2012). β-turn tendency in *N*-methylated peptides with dehydrophenylalanine residue: DFT study. Biopolymers.

[61] Wang Z-X, Duan Y (2004). Solvation effects on alanine dipeptide: a MP2/cc-pVTZ//MP2/6-31G** study of (Φ, Ψ) energy maps and conformers in the gas phase, ether, and water. J Comput Chem.

[62] Drozdov AN, Grossfield A, Pappu RV (2004). Role of solvent in determining conformational preferences of alanine dipeptide in water. J Am Chem Soc.

[63] Smith PE (1999). The alanine dipeptide free energy surface in solution. J Chem Phys.

[64] Ilawe NV, Raeber AE, Schweitzer-Stenner R, Toal SE, Wong BM (2015). Assessing backbone solvation effects in the conformational propensities of amino acid residues in unfolded peptides. Phys Chem Chem Phys.

[65] Lanza G, Chiacchio MA (2015). Interfacial water at the trialanine hydrophilic surface: A DFT electronic structure and bottom-up investigation. Phys Chem Chem Phys.

[66] Scheiner S, Kar T (2007). Underlying source of the relation between polypeptide conformation and strength of NH⋯O hydrogen bonds. J Mol Struct.

[67] Scheiner S (2007). The strength with which a peptide group can form a hydrogen bond varies with the internal conformation of the polypeptide chain. J Phys Chem B.

[68] Baldauf C, Hofmann H-J (2012). Ab initio MO theory—an important tool in foldamer research: prediction of helices in oligomers of ω-amino acids. Helv Chim Acta.

[69] Marianski M, Asensio A, Dannenberg JJ (2012). Comparison of some dispersion-corrected and traditional functionals as applied to peptides and conformations of cyclohexane derivatives. J Chem Phys.

