# Supplementary material for: The influence of solvent on conformational properties of peptides with Aib residue—a DFT study
Source: J Mol Model. 2017 Nov 21;23(12):349. doi: 10.1007/s00894-017-3508-4 (PMC5698364; doi:10.1007/s00894-017-3508-4)
Supplement: Supplementary file 1 — (PDF 3.71 mb). [file 894_2017_3508_MOESM1_ESM.pdf]

Supplementary material for:

The influence of solvent on conformational properties of peptides with Aib residue. DFT study

Roksana Wałęsa, Małgorzata A. Broda*

Faculty of Chemistry, University of Opole, Oleska 48, 45-052 Opole, Poland

Table S1. Structural parameters for the internal X–H···A interactions in the B3LYP/6–31++G** M06-2X/6–31++G** and MP2/6–31++G** geometries of molecules **1** and **2**

Table S2. Structural parameters for the internal C=O dipole attractions in the studied molecules **1** and **2**

Figure S1. The potential energy surfaces E=*f*(ϕ, ψ) of Ac-Aib-NHMe (**1**) in chloroform and in water calculated by the M06-2X/6-31++G(d,p) method with SMD solvent model. Energy contours are drawn every 1 kcal/mol. Local minima are represented by ♦ and minima found on the upper halves of the maps are described by the general short hand letter notation.

Figure S2. The potential energy surfaces E=*f*(ϕ, ψ) of Ac-Aib-NMe_2 (**2**) in chloroform and in water calculated by the M06-2X/6-31++G(d,p) method with SMD solvent model. Energy contours are drawn every 1 kcal/mol. Local minima are represented by ♦ and minima found on the upper halves of the maps are described by the general short hand letter notation.

Figure S3. The potential energy surfaces E=*f*(ϕ, ψ) of Ac-Aib-NHMe (**1**) *in vacuo* (A) calculated by the B3LYP/6-31++G(d,p) and by the MP2/6-31++G(d,p) methods; in chloroform and in water (B) calculated by the B3LYP/6-31++G(d,p) method with PCM or SMD solvent models. Energy contours are drawn every 1 kcal/mol. Local minima are represented by ♦ and minima found on the upper halves of the maps are described by the general short hand letter notation.

Figure S4. The potential energy surfaces E=*f*(ϕ, ψ) of Ac-Aib-NMe_2 (**2**) *in vacuo* (A) calculated by the B3LYP/6-31++G(d,p) and by the MP2/6-31++G(d,p) methods; in chloroform and in water (B) calculated by the B3LYP/6-31++G(d,p) method with PCM or SMD solvent models. Energy contours are drawn every 1 kcal/mol. Local minima are represented by ♦ and minima found on the upper halves of the maps are described by the general short hand letter notation.

**Table S1**. Structural parameters for the internal X–H···A interactions in the B3LYP, M06-2X and MP2 geometries of molecules **1** and **2**								
conformer	NH···O or NH···N				CH···O			
	H···O(N)	N···O(N)	∠N–H···O(N)	∠C=O···H	H···O	C···O	∠C–H···O	∠C=O···H
**gas phase**								
B3LYP/6-31++G(d,p)								
Ac-Aib-NHMe (**1**)								
C	1.90	2.83	151.8	103.6	2.46	3.09	115.5	96.9
E	2.03	2.59	112.5	87.5	2.45<br>2.45	3.12<br>3.12	117.8<br>117.8	94.6<br>94.6
F	-	-	-	-	2.45	3.07	114.5	97.5
A	2.32	2.78	106.9 (NHN)	-	2.47	3.10	115.7	93.3
D	2.40	2.73	98.1 (NHN)	-	2.39<br>2.72<br>3.00	2.99<br>3.30<br>3.23	113.3<br>113.3<br>92.2	102.8<br>86.7<br>62.0
Ac-Aib-NMe$_2$ (**2**)								
E	1.94	2.53	113.9	90.9	2.20<br>2.40<br>2.40	2.69<br>3.10<br>3.10	104.7<br>120.7<br>120.7	87.6<br>93.1<br>93.1
A	-	-	-	-	2.22	2.71	104.7	86.6
D	-	-	-	-	2.33<br>2.42	2.66<br>3.03	95.5<br>113.5	85.1<br>102.3
D$_2$	-	-	-	-	2.24<br>2.26	2.48<br>3.30	103.5<br>158.6	86.3<br>85.6
F	-	-	-	-	2.36<br>2.36<br>2.43	3.27<br>2.67<br>2.79	140.1<br>93.6<br>97.5	128.9<br>83.4<br>85.4
M06-2X/6-31++G(d,p)								
Ac-Aib-NHMe (1)								
C	1.93	2.85	149.1	103.5	2.39	3.01	115.0	99.1
E	2.05	2.59	110.9	87.5	2.40<br>2.40	3.06<br>3.06	117.9<br>117.9	95.4<br>95.5
F	-	-	-	-	2.44	3.05	113.6	96.7
A	2.32	2.77	105.7 (NHN)	-	2.46	3.08	114.9	92.4
D	2.39	2.71	97.1 (NHN)	-	2.36<br>2.61<br>2.58	2.95<br>3.21<br>2.78	112.2<br>114.2<br>93.0	103.7<br>88.7<br>61.6
Ac-Aib-NMe$_2$ (2)								
E	1.76	2.53	112.0	90.6	2.21<br>2.32<br>2.37	2.68<br>3.05<br>3.05	103.9<br>122.5<br>119.1	87.1<br>92.7<br>94.2
A	-	-	-	-	2.23	2.70	103.4	86.3
D	-	-	-	-	2.36<br>2.39	2.64<br>2.99	92.8<br>113.1	84.3<br>102.9
F	-	-	-	-	2.38<br>2.32<br>2.44	2.64<br>3.18<br>2.80	91.6<br>134.1<br>97.5	82.6<br>131.3<br>83.8
D$_2$	-	-	-	-	2.27<br>2.25	2.70<br>3.24	100.7<br>149.1	85.1<br>84.6
MP2/6-31++G(d,p)								
Ac-Aib-NHMe (1)								
C	1.89	2.83	152.2	102.0	2.43	3.06	115.7	97.3
E	2.04	2.59	111.7	87.1	2.41	3.08	118.5	94.4

F	-	-	-	-	2.41<br>2.47	3.08<br>3.07	118.5<br>113.5	94.4<br>96.4
A	2.29	2.76	106.7<br>(NHN)	-	2.50	3.11	114.4	90.8
D	2.52	2.69	97.4 (NHN)	-	2.96<br>2.38<br>2.62	3.21<br>2.97<br>3.23	93.4<br>113.0<br>114.9	62.0<br>102.9<br>87.1
Ac-Aib-NMe_2 (2)								
E	1.95	2.52	113.1	90.4	2.20<br>2.36<br>2.36	2.70<br>3.07<br>3.07	105.2<br>121.1<br>121.1	86.9<br>92.9<br>92.9
A	-	-	-	-	2.32	2.66	95.7	85.3
D	-	-	-	-	2.39<br>2.41	2.65<br>3.01	91.2<br>113.3	83.2<br>102.1
F	-	-	-	-	2.37<br>2.46<br>2.40	3.21<br>2.82<br>2.65	133.4<br>97.6<br>91.2	130.4<br>82.6<br>82.1
D_2	-	-	-	-	2.35<br>2.25	2.68<br>3.25	95.7<br>153.2	83.3<br>83.9
M06-2X/6-31++G(d,p) SMD chloroform								
Ac-Aib-NHMe (1)								
A	2.34	2.76	104.1 (NHN)	-	2.50	3.09	113.1	93.1
C	1.92	2.85	150.5	102.0	2.41	3.02	113.6	99.4
E	2.06	2.59	110.6	87.7	2.43<br>2.45	3.09<br>3.09	117.7<br>116.5	95.1<br>94.8
F	-	-	-	-	-	-	-	-
D	2.41	2.73	97.3 (NHN)	-	2.40<br>2.63<br>2.99	2.96<br>3.23<br>3.23	110.3<br>114.3<br>92.8	103.6<br>88.0<br>60.0
Ac-Aib-NMe_2 (2)								
A	-	-	-	-	2.33	2.68	96.1	83.4
E	1.98	2.53	111.9	90.4	2.39<br>2.40	3.07<br>3.08	119.5<br>118.9	93.9<br>93.5
D	-	-	-	-	2.44	2.99	109.9	103.2
D_2	-	-	-	-	2.31	3.28	148.2	82.5
F	-	-	-	-	2.41<br>2.46	3.20<br>2.80	128.8<br>96.4	128.2<br>83.7
M06-2X/6-31++G(d,p) SMD water								
Ac-Aib-NHMe (1)								
F	-	-	-	-	-	-	-	-
A	2.41	2.79	101.2 (NHN)	-	-	-	-	-
E	2.06	2.59	110.4	87.7	2.47<br>2.48	3.11<br>3.19	116.8<br>116.3	95.2<br>94.8
C	1.94	2.87	151.8	99.0	2.42	3.03	113.3	99.2
D	2.43	2.75	97.5 (NHN)	-	2.46<br>2.65<br>2.94	2.97<br>3.26<br>3.22	107.1<br>114.7<br>94.5	103.3<br>87.2<br>60.6
Ac-Aib-NMe_2 (2)								
A	-	-	-	-	-	-	-	-
E	1.99	2.54	111.1	90.0	2.48	2.96	105.2	103.6
F	-	-	-	-	-	-	-	-
D_2	-	-	-	-	2.35	3.29	143.1	81.7
D	-	-	-	-	2.49	2.96	105.2	103.6

M06-2X/6-31++G(d,p) PCM chloroform								
Ac-Aib-NHMe (1)								
C	1.92	2.85	150.1	103.2	2.39	3.01	114.3	99.3
A	2.34	2.76	104.1 (NHN)	-	2.48	3.09	113.9	92.8
E	2.05	2.59	110.9	87.7	2.42<br>2.42	3.08<br>3.08	117.6<br>117.6	95.1<br>95.1
F	-	-	-	-	2.50	3.09	112.2	95.4
D	2.40	2.72	97.6 (NHN)	-	2.38<br>2.62<br>2.97	2.96<br>3.23<br>3.22	111.1<br>114.7<br>93.2	103.6<br>88.2<br>61.0
Ac-Aib-NMe$_2$ (2)								
A	-	-	-	-	2.24	2.71	104.0	85.8
E	1.97	2.53	112.2	90.5	2.38<br>2.37	3.07<br>3.08	119.0<br>120.9	94.4<br>92.8
F	-	-	-	-	2.37<br>2.40<br>2.45	3.19<br>2.65<br>2.80	131.5<br>90.8<br>96.9	129.7<br>82.3<br>83.8
D$_2$	-	-	-	-	2.29<br>2.33	3.26<br>2.69	147.8<br>97.1	83.2<br>84.0
D	-	-	-	-	2.41<br>2.39	2.99<br>2.65	112.1<br>91.5	102.8<br>83.6
M06-2X/6-31++G(d,p) PCM water								
Ac-Aib-NHMe (1)								
A	2.36	2.77	102.7 (NHN)	-	2.50	3.10	113.4	92.7
F	-	-	-	-	-	-	-	-
C	1.91	2.84	151.3	102.1	2.40	3.01	113.9	99.2
E	2.05	2.59	111.0	87.8	2.44<br>2.43	3.09<br>3.09	117.3<br>117.4	94.9<br>94.9
D	2.39	2.73	98.1 (NHN)	-	2.39<br>2.63<br>2.96	2.95<br>3.24<br>3.21	110.5<br>114.6<br>93.5	103.5<br>87.8<br>61.2
Ac-Aib-NMe$_2$ (2)								
A	-	-	-	-	2.25	2.72	103.6	85.7
D	-	-	-	-	2.41<br>2.42	2.65<br>3.00	90.6<br>111.8	83.1<br>102.6
D$_2$	-	-	-	-	2.32<br>2.37	3.28	146.4<br>94.5	82.2<br>83.2
F	-	-	-	-	2.42<br>2.46<br>2.40	3.22<br>2.81<br>2.65	129.5<br>96.5<br>90.7	128.1<br>83.5<br>82.2
E	1.97	2.53	112.3	90.5	2.38<br>2.40	3.08<br>3.08	120.3<br>118.9	93.0<br>94.1
B3LYP/6-31++G(d,p) SMD chloroform								
Ac-Aib-NHMe (1)								
E	2.03	2.60	112.5	87.7	2.49	3.14	117.4	94.3
C	1.89	2.83	153.0	102.5	2.48	3.10	114.3	97.1
A	2.34	2.78	105.2	-	-	-	-	-
F	-	-	-	-	-	-	-	-
D	2.42	2.75	97.8 (NHN)	-	2.43<br>2.73<br>3.03	3.00<br>3.32<br>3.26	111.4<br>113.4<br>91.9	102.3<br>86.3<br>60.1
Ac-Aib-NMe$_2$ (2)								
E	1.94	2.53	114.1	90.6	2.48	3.14	118.8	92.3

					2.44	3.13	120.1	92.8
A	-	-	-	-	2.40	2.66	91.7	83.4
D	-	-	-	-	2.42<br>2.46	2.66<br>3.02	90.3<br>110.6	83.6<br>102.6
D_2	-	-	-	-	2.37<br>2.38	3.39<br>2.68	156.7<br>93.6	82.3<br>83.7
F	-	-	-	-	2.49<br>2.41<br>2.47	3.33<br>2.66<br>2.81	133.2<br>91.0<br>96.5	126.7<br>82.6<br>84.2
B3LYP/6-31++G(d,p) SMD water								
Ac-Aib-NHMe (1)								
F	-	-	-	-	-	-	-	-
A	2.42	2.81	102.0 (NHM)	-	-	-	-	-
E	2.04	2.60	112.3	87.7	-	-	-	-
B	1.90	2.85	154.0	100.2	2.50	3.11	113.8	96.8
D	2.45	2.78	97.7 (NHN)	-	2.50<br>2.73<br>3.00	3.02<br>3.33<br>3.26	108.3<br>114.0<br>93.3	101.9<br>86.2<br>59.9
Ac-Aib-NMe_2 (2)								
A	-	-	-	-	-	-	-	-
E	1.95	2.54	113.9	90.7	2.49<br>2.49	3.17<br>3.15	118.9<br>118.1	91.5<br>92.7
D	-	-	-	-	-	-	-	-
F	-	-	-	-	2.40	2.66	90.9	83.1
D_2	-	-	-	-	2.45	3.45	153.4	79.8
B3LYP/6-31++G(d,p) PCM chloroform								
Ac-Aib-NHMe (1)								
C	1.89	2.83	152.9	103.1	2.47	3.09	114.9	97.0
E	2.02	2.59	112.9	87.7	2.49<br>2.49	3.14<br>3.14	117.2<br>117.2	94.2<br>94.2
A	2.33	2.78	105.5<br>(NHN)	-	2.50	3.12	114.6	93.5
F	-	-	-	-	-	-	-	-
D	2.41	2.74	98.3(NHN)	-	2.41<br>2.73<br>3.01	3.00<br>3.32<br>3.24	112.2<br>113.5<br>92.3	102.5<br>86.3<br>61.1
Ac-Aib-NMe_2 (2)								
E	1.94	2.53	114.3	90.7	2.34<br>2.44<br>2.42	2.66<br>3.13<br>3.12	94.8<br>119.9<br>120.5	85.1<br>92.2<br>93.0
A	-	-	-	-	2.26	2.71	102.6	85.7
D	-	-	-	-	2.37<br>2.44	2.67<br>3.04	93.5<br>112.9	84.2<br>101.9
D_2	-	-	-	-	2.32<br>2.34	2.70<br>3.36	98.1<br>156.2	84.7<br>82.8
F	-	-	-	-	2.46<br>2.38<br>2.46	3.32<br>2.66<br>2.81	135.4<br>92.5<br>96.9	127.7<br>82.9<br>84.4
B3LYP/6-31++G(d,p) PCM water								
Ac-Aib-NHMe (1)								
A	2.37	2.79	103.8 (NHN)	-	-	-	-	-
C	1.88	2.83	153.6	102.6	2.47	3.09	114.7	96.9
E	2.02	2.59	113.0	87.8	-	-	-	-

F	-	-	-	-	-	-	-	-
D	2.40	2.74	98.8 (NHN)	-	2.42<br>2.74<br>3.00	3.00<br>3.33<br>2.64	111.6<br>113.5<br>92.7	102.4<br>86.0<br>61.6
Ac-Aib-NMe$_2$ (2)								
A	-	-	-	-	2.34	2.68	96.0	85.2
E	1.94	2.53	114.5	90.6	2.46<br>2.44	3.14<br>3.13	119.3<br>119.3	92.3<br>92.3
D	-	-	-	-	2.40<br>2.46	2.67<br>3.04	92.1<br>112.5	83.6<br>101.7
D$_2$	-	-	-	-	2.36<br>2.40	2.69<br>3.40	95.7<br>154.3	83.8<br>81.1
F	-	-	-	-	2.39<br>2.48	2.66<br>2.82	92.0<br>96.5	82.7<br>83.8

Data presented only for the X–H···A contacts (X = N, C; A = O, N) in which H···A < 2.5 Å and <X–H···A > 90° according to Ref. [47]. Distances are given in (Å). Angles are given in (°).

Table S2. Structural parameters for the internal C=O dipole attractions in the studied molecules **1** and **2**

conformer	$C^{C}\cdots O^{N}$	$C^{N}\cdots O^{C}$	C···C	O···O	$\angle(C{=}O)^{N}\cdots C^{C}$	$\angle O^{N}\cdots(C{=}O)^{C}$	$\angle C^{N}\cdots(O{=}C)^{C}$	$\angle O^{C}\cdots(C{=}O)^{N}$
gas phase								
B3LYP/6-31G++(d,p)								
				Ac-Aib-NHMe **(1)**				
C	3.38	4.44	3.30	4.60	75.5	172.3	18.6	89.8
F	3.00	3.39	2.99	3.62	77.9	110.7	60.7	90.5
A	3.18	4.29	3.23	4.04	81.0	126.9	25.4	70.0
				Ac-Aib-NMe$_2$ **(2)**				
A	4.00	2.92	3.10	3.54	86.7	110.3	37.3	60.1
F	3.46	3.01	3.05	3.75	77.0	114.3	60.0	93.4
M06-2X/6-31G++(d,p)								
				Ac-Aib-NHMe **(1)**				
C	3.37	4.41	3.27	4.59	75.0	172.1	19.2	90.5
F	2.81	3.22	2.87	3.37	80.7	106.9	62.7	86.3
A	3.01	4.16	3.12	3.82	83.8	123.2	27.2	65.5
				Ac-Aib-NMe$_2$ **(2)**				
A	3.89	2.77	3.00	3.39	88.7	109.6	37.0	57.1
F	2.86	3.27	3.05	3.14	68.9	92.0	87.3	73.3
MP2/6-31G++(d,p)								
				Ac-Aib-NHMe **(1)**				
C	3.37	4.44	3.28	4.62	74.8	173.8	17.7	90.2
F	2.81	3.18	2.86	3.36	79.7	105.3	63.8	87.0
A	2.96	4.11	3.09	3.72	84.3	119.1	29.4	63.3
				Ac-Aib-NMe$_2$ **(2)**				
A	3.87	2.75	3.00	3.34	89.0	107.8	38.3	56.2
F	3.20	2.82	2.87	3.41	79.7	107.4	63.7	88.8
M06-2X/6-31++G(d,p) SMD chloroform								
				Ac-Aib-NHMe **(1)**				
A	2.95	4.06	3.07	3.67	83.7	116.7	30.7	63.0
C	3.38	4.43	3.28	4.60	74.8	174.1	17.9	90.3
F	2.81	3.16	2.90	3.25	81.7	99.5	66.8	83.2
				Ac-Aib-NMe$_2$ **(2)**				
A	2.85	3.94	3.02	3.47	85.9	110.3	35.4	59.2
F	2.81	3.28	2.91	3.40	82.4	107.9	62.1	84.7
M06-2X/6-31++G(d,p) SMD water								
				Ac-Aib-NHMe **(1)**				
F	2.79	3.12	2.92	3.13	83.3	93.9	69.1	78.9
A	2.89	3.98	3.02	3.55	83.9	112.2	32.9	60.8
C	3.39	4.47	3.29	4.62	74.9	178.4	15.0	89.3
				Ac-Aib-NMe$_2$ **(2)**				
A	2.78	3.82	2.96	3.30	86.1	103.6	39.1	56.2
F	2.77	3.21	2.92	3.25	84.2	101.6	65.2	80.8
M06-2X/6-31++G(d,p) PCM chloroform								
				Ac-Aib-NHMe **(1)**				
C	3.37	4.41	3.27	4.59	75.0	171.9	19.3	90.5
A	2.94	4.06	3.07	3.67	84.1	116.8	30.6	62.7
F	2.80	3.17	2.90	3.26	81.9	100.6	66.1	83.2
				Ac-Aib-NMe$_2$ **(2)**				
A	2.81	3.90	3.00	3.41	87.1	108.8	36.7	57.8
F	2.81	3.29	2.92	3.41	82.4	108.4	61.9	85.0
M06-2X/6-31++G(d,p) PCM water								
				Ac-Aib-NHMe **(1)**				
A	2.81	3.89	3.00	3.39	86.6	107.6	37.2	57.6

F	2.79	3.29	2.92	3.37	83.5	107.1	62.1	82.9
Ac-Aib-NMe_2 **(2)**								
A	2.81	3.89	3.00	3.39	86.6	107.6	37.2	57.6
F	2.79	3.29	2.92	3.37	83.5	107.1	62.1	82.9
B3LYP/6-31++G(d,p) SMD chloroform								
Ac-Aib-NHMe **(1)**								
C	3.38	4.45	3.30	4.62	75.3	173.4	17.7	89.6
A	3.11	4.19	3.16	3.88	81.1	120.4	28.7	67.2
F	2.97	3.28	3.01	3.43	79.5	100.8	66.3	86.1
Ac-Aib-NMe_2 **(2)**								
A	2.99	4.01	3.12	3.59	84.3	108.9	37.5	61.4
F	2.97	3.40	3.01	3.59	79.9	109.8	61.4	88.3
B3LYP/6-31++G(d,p) SMD water								
Ac-Aib-NHMe **(1)**								
F	2.93	3.23	3.01	3.28	81.5	95.0	68.6	81.2
A	3.03	4.09	3.11	3.72	81.6	114.1	32.2	64.2
B	3.39	4.50	3.31	4.64	75.4	177.2	15.0	88.7
Ac-Aib-NMe_2 **(2)**								
A	2.99	3.96	3.09	3.52	82.8	104.8	39.2	60.8
F	2.89	3.23	2.99	3.33	81.8	98.7	67.6	83.3
B3LYP/6-31G++(d,p) PCM chloroform								
Ac-Aib-NHMe **(1)**								
C	3.38	4.45	3.30	4.61	75.5	172.7	18.3	89.5
A	3.12	4.20	3.17	3.89	81.2	120.5	28.8	67.3
F	2.97	3.29	3.00	3.44	79.5	101.8	65.7	101.8
Ac-Aib-NMe_2 **(2)**								
A	4.00	2.96	3.11	3.56	84.9	108.8	37.5	60.7
F	3.42	2.99	3.02	3.62	79.5	110.9	60.9	89.1
B3LYP/6-31G++(d,p) PCM water								
Ac-Aib-NHMe **(1)**								
A	3.05	4.12	3.13	3.76	81.7	115.7	31.4	64.9
C	3.38	4.46	3.30	4.61	75.6	173.6	17.6	89.2
F	2.95	3.26	3.01	3.35	80.9	97.7	67.5	97.7
Ac-Aib-NMe_2 **(2)**								
A	2.92	3.91	3.09	3.43	85.7	103.6	41.1	58.5
F	3.39	2.93	3.01	3.51	81.5	107.8	61.9	85.5

Data presented only for C=O dipole attractions in which C···O <3.6 Å acc. to ref. [48]. Distances are given in (Å). Angles are given in (°). ^{N},^{C} — ascribe the structural parameters to the N –terminal and C–terminal, respectively.

\

chloroform   water
SMD /M06-2X   SMD/M06-2X

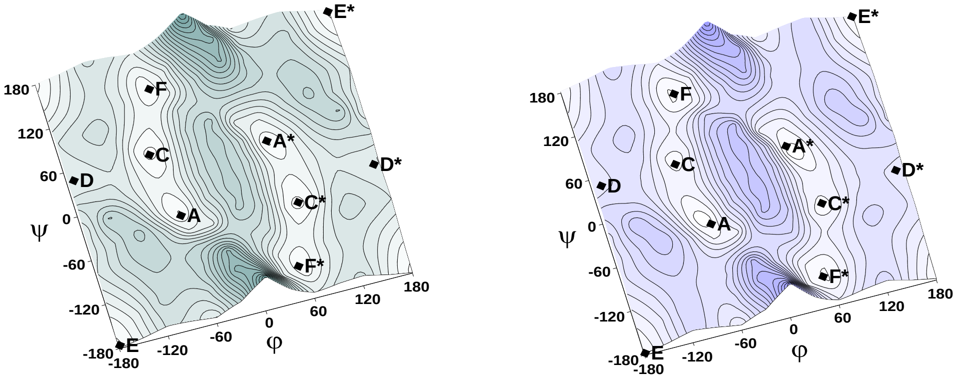

Figure S1. Ac-Aib-NHMe (**1**)

chloroform   water
SMD /M06-2X   SMD/M06-2X

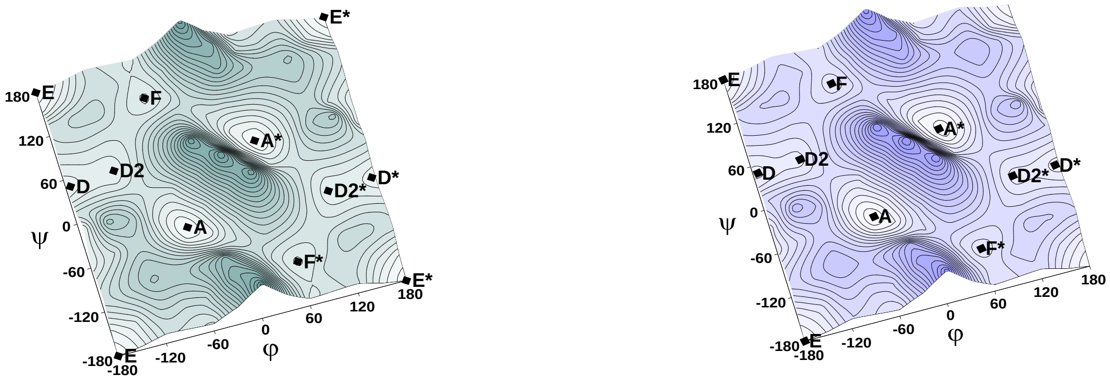

Figure S2. Ac-Aib-NMe$_2$ (**2**)

(A) gas phase

B3LYP

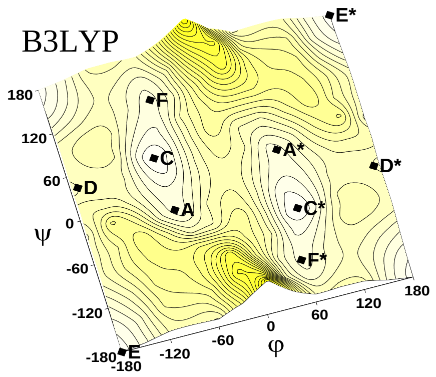

MP2

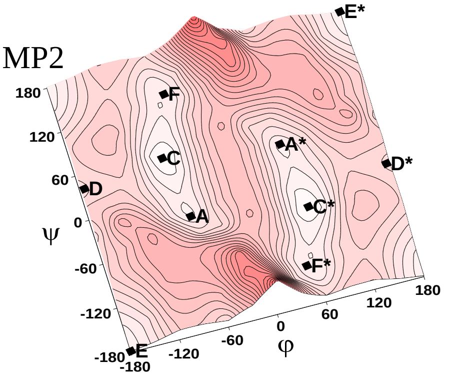

(B) solvent environment

chloroform

SMD/M06-2X

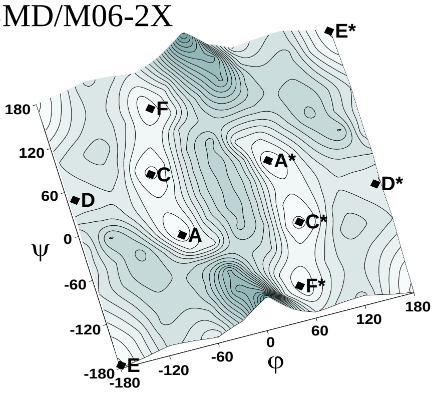

water

SMD/M06-2X

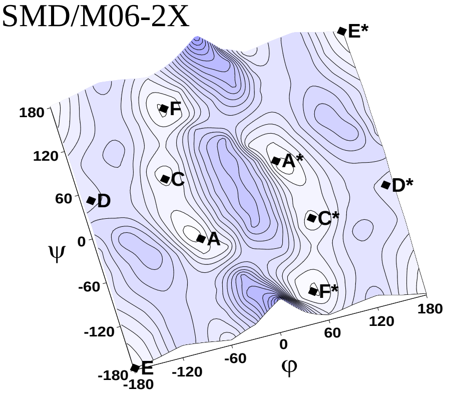

PCM/B3LYP

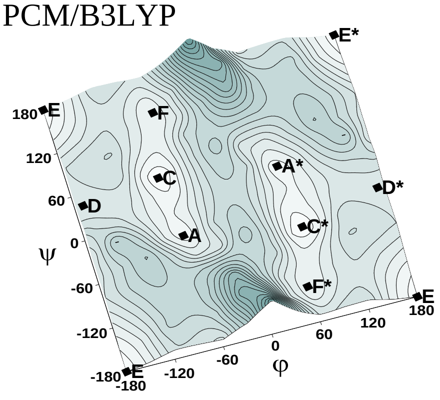

PCM/B3LYP

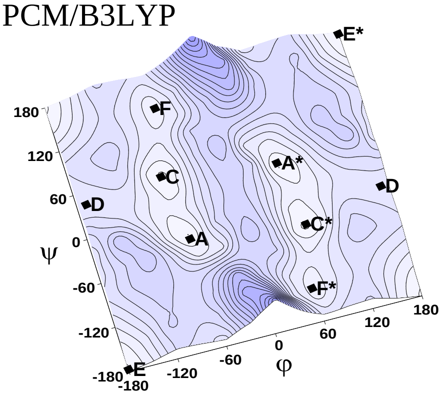

SMD/B3LYP

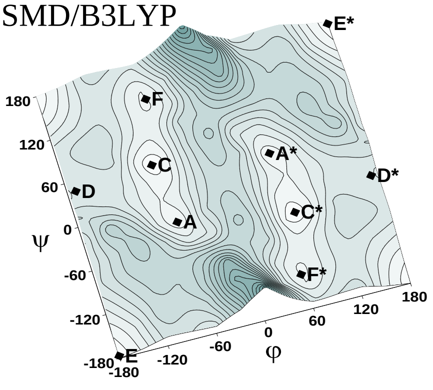

SMD/B3LYP

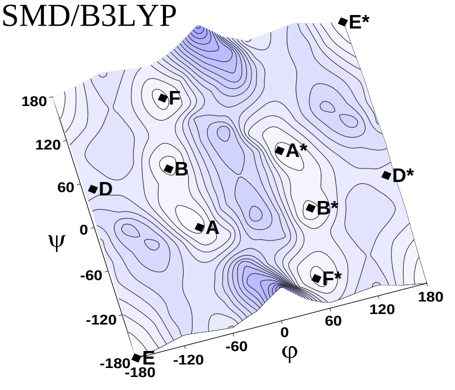

Figure S3. Ac-Aib-NHMe

(A) gas phase

B3LYP

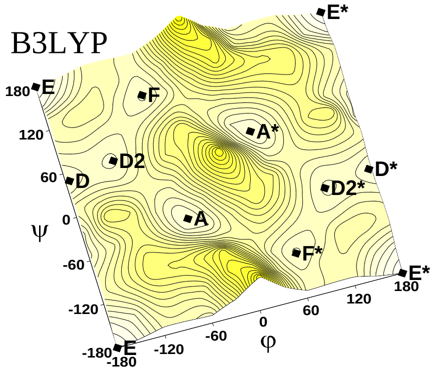

MP2

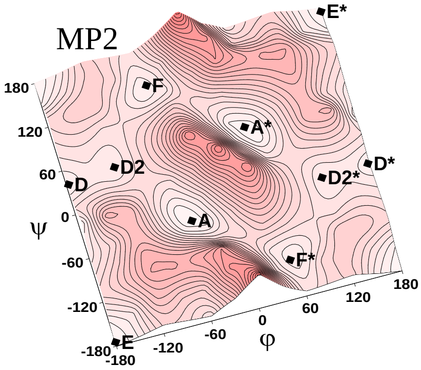

(B) solvent environment

chloroform
SMD /M06-2X

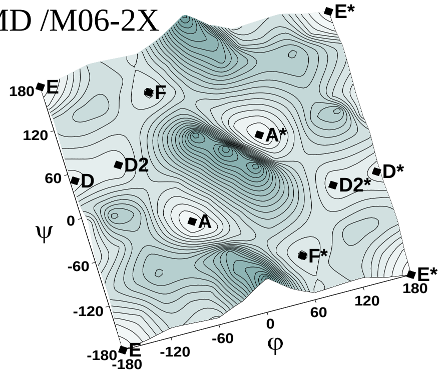

water
SMD/M06-2X

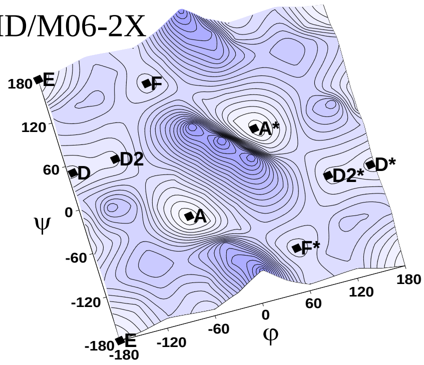

PCM/B3LYP

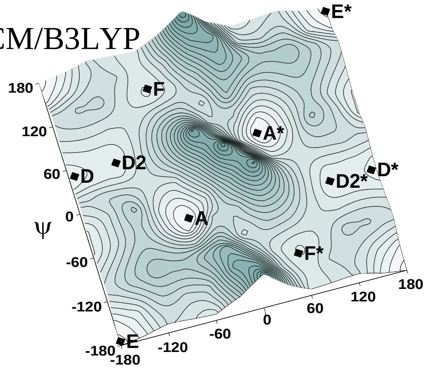

PCM/B3LYP

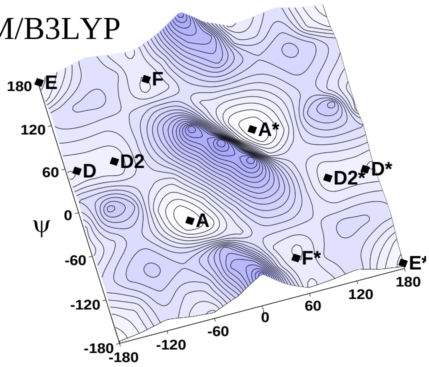

SMD/B3LYP

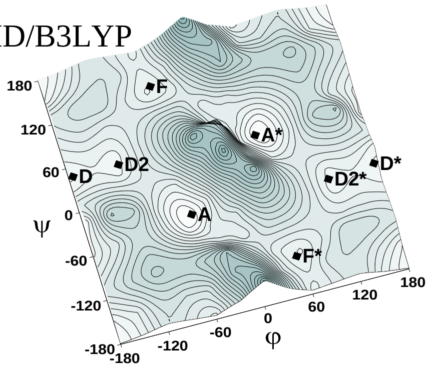

SMD/B3LYP

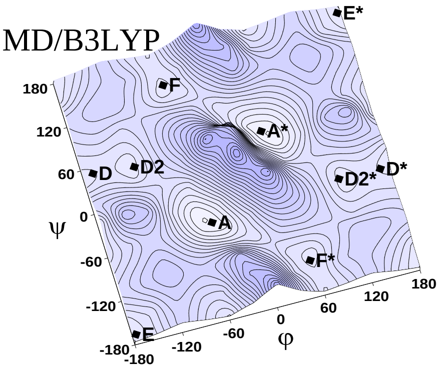

Figure S4. Ac-Aib-NMe_2
